# Can Brief, Daily Training Using a Mobile App Help Change Maladaptive Beliefs? Crossover Randomized Controlled Trial

**DOI:** 10.2196/11443

**Published:** 2019-02-13

**Authors:** María Roncero, Amparo Belloch, Guy Doron

**Affiliations:** 1 Research and Treatment Unit for Obsessive-Compulsive and Related Disorders, Department of Personality Faculty of Psychology University of Valencia Valencia Spain; 2 Baruch Ivcher School of Psychology Interdisciplinary Center Herzliya Herzliya Israel

**Keywords:** obsessive compulsive disorder, cognitive therapy, maladaptive beliefs, mobile apps, relationships

## Abstract

**Background:**

Obsessive-compulsive disorder (OCD) is a disabling condition with a wide variety of clinical presentations including contamination fears, fear of harm, and relationship-related obsessions. Cognitive behavioral models of OCD suggest that OC symptoms result from catastrophic misinterpretations of commonly occurring intrusive experiences and associated dysfunctional strategies used to manage them. OCD-related maladaptive beliefs including inflated responsibility, importance and control of thoughts, perfectionism, and intolerance for uncertainty increase the likelihood of such misinterpretations.

**Objective:**

Considering accumulating evidence suggesting that mobile health (mHealth) apps based on cognitive-behavioral principles may lead to significant reductions in psychopathological symptoms, we assessed the effectiveness of a novel cognitive training app (GGRO) designed to challenge OCD-related beliefs.

**Methods:**

A total of 97 students were randomized to groups undertaking immediate-use (iApp) or delayed use (dApp) of GGRO. All participants were requested to complete Web-based assessments, with questionnaires relating to maladaptive beliefs, mood, and OC symptoms at baseline (T1), 15 days from baseline (T2), and 30 days from baseline (T3). Participants in iApp group started using the app at baseline and continued using the app for 15 consecutive days. They were then requested to stop using the app until T3. Participants in the dApp group were requested to wait for 15 days and only then start using the app (crossover) for 15 consecutive days.

**Results:**

All participants used the app for a mean of 14.07 (SD 1.41) days with 2.94 levels per day. Consistent with previous findings, app use was associated with medium-large effect size reductions in both iApp (n=51) and dApp (n=46) groups. In the iApp group, all effects remained significant during the 15 days of follow-up. Analyses focusing on the first two assessment occasions revealed significant treatment × repeated measures interactions on maladaptive beliefs, several OC symptom measures, and self-esteem.

**Conclusions:**

This study provides further evidence for the efficacy of GGRO as a mobile-delivered training exercise that is useful for reducing OCD-related beliefs and symptoms.

**Trial Registration:**

ClinicalTrials.gov NCT03571464; https://clinicaltrials.gov/ct2/show/NCT03571464 (Archived by WebCite at http://www.webcitation.org/7675sYPsH)

## Introduction

Obsessive-compulsive disorder (OCD) is a disabling disorder that causes impairment in multiple areas of patients’ lives [1,2]. OCD is characterized by the presence of repetitive unwanted and disturbing intrusive thoughts, images or urges (obsessions), or ritualistic and repetitive acts (compulsions) [3]. The content of OCD is heterogenic, comprising themes as scrupulosity [4], repugnant obsessions [5], moral and physical contamination fears [6], cleaning compulsions, obsessional doubts, and relationship-related obsessions [7].

Cognitive behavioral therapy (CBT) combined with exposure and ritual prevention is the first choice of psychological treatment recognized by the National Institute for Clinical Excellence [8]. CBT models of OCD postulate that catastrophic misinterpretation of intrusive thoughts or images and urges and the use of counterproductive cognitive and behavioral strategies to manage them lead to their escalation into chronic obsessions [9-11]. A number of maladaptive beliefs have been found to be associated with this catastrophic misinterpretation: inflated responsibility, overimportance of thoughts, desire to control one’s thoughts, overestimation of threat, and need for certainty and perfectionism [12,13].

Many individuals, however, have great difficulties in accessing CBT therapy, either because of their high cost, the stigma associated with treatment, or the lack of available trained professionals [14,15]. Information and Communication Technologies including mobile apps and internet-based interventions have been suggested to increase accessibility and availability of CBT-based interventions [16-18]. Such alternative CBT-delivery systems are consistent with the stepped-care approach for OCD [8]. Clients with OCD may begin with low-intensity interventions (eg, self-help materials) and, if needed, gradually receive more intense and expert interventions [19].

Information and Communication Technologies have been implemented to a significantly smaller extent in the treatment or prevention of OCD symptoms than in that of other mental disorders. Most studies assessed the efficacy of video-conference or telephone therapy used in exposure and response prevention [20-22]. In addition, most existing CBT-based mobile apps translate internet-delivered desktop treatment programs into mobile apps without exploiting the special advantages of the mobile app platform. These programs often have a long duration (eg, more than 20 min per interaction) and involve tasks requiring high internal motivation, a long attention span, and high persistence from users (eg, enter a significant amount of free text) [23].

Recently, an exploratory study evaluated a brief, game-like training exercise for challenging OCD beliefs delivered via a mobile app platform named “GGRO - GG relationship doubt and obsessions V1.1” [24]. GGRO is one of various mobile apps designed by GGApps (Herzliya, Israel) to challenge beliefs associated with a range of psychological difficulties (eg, depression, body image distress, and low self-esteem). GGRO was specifically designed to challenge maladaptive beliefs that underlie common OCD symptoms (eg, contamination and repugnant thoughts) as well as relationship obsessions (eg, obsessive preoccupations regarding the suitability of the relationship or the relationship partners) [7,12,25]. The platform was designed to help users learn to respond to statements that challenge OCD-related beliefs by embracing them (ie, pulling them down toward themselves) and rejecting statements that are consistent with beliefs underlying OCD symptoms and low self-esteem (ie, throwing them upward, away from themselves; see Methods section). Following CBT principles, statements challenging OCD-related beliefs include alternative, more adaptive interpretations of thoughts, emotions, and events as well as statements encouraging approach behavioral strategies (eg, tolerance of negative feelings and acceptance of thoughts). Increasing accessibility to such statements is expected to reduce adherence to OCD-related beliefs and associated symptoms.

The results of this study, which included 20 participants from a nonclinical population, suggested that training for 3 min a day for a period of 15 days was associated with significant, large effect-size reductions in the levels of OCD-related beliefs measured by the Obsessive Beliefs Questionnaire (OBQ) - Short form [24]. Participants also showed significant pre-post training decreases in the levels of OCD symptoms, measured by the Obsessive-Compulsive Inventory - Reduced (OCI-R) [26], including relationship-related OCD symptoms measured using the Relationship Obsessive-Compulsive Inventory (ROCI) [27] and the Partner-Related Obsessive-Compulsive Symptoms Inventory (PROCSI; R Moulding, GD, unpublished data, 2019). Moreover, pre-post changes in the levels of OCD-beliefs were associated with a reduction in OCD symptom levels.

**Figure 1:**
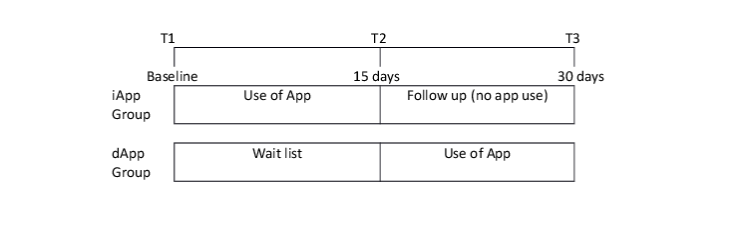
Study design with both groups. iApp: immediate-use App; dApp: delayed-use App.

The aim of this study was to further evaluate the efficacy of GGRO in reducing OCD-related maladaptive beliefs and OCD symptoms. Specifically, a randomized controlled trial with crossover design was carried out in a nonclinical student population to assess pre-post changes in the levels of OCD-related maladaptive beliefs and OCD symptoms, including relationship OCD (ROCD) symptoms, self-esteem, and depression symptoms following 15 days of GGRO use. Our main hypothesis was that students using GGRO immediately following baseline assessment (immediate-use App group, iApp) would exhibit greater declines in obsessive compulsive–related beliefs than students who did not use GGRO in this phase of the study (delayed-use App group, dApp; Figure 1). Consistent with previous research showing an association between OCD symptoms and self-esteem [28,29], we expected a decrease in OCD and ROCD symptoms and an increase in self-esteem in students in the iApp group relative to the students in the dApp control group. Following crossover (T2), we expected that user gains in the iApp group would be maintained in T3. In this phase, we anticipated that students starting to use GGRO (dApp) would show statistically significant reductions in OCD-related maladaptive beliefs and symptoms and an increase in self-esteem from T2 to T3 assessments. Consistent with a previous study using GGRO in a student population [24], we did not expect a significant reduction in depression symptoms.

## Methods

### Participants

Participants were recruited from the University of Valencia during the first semester of the 2016/2017 course from nine classes at the Psychology Faculty. The students were invited to voluntarily participate in a study about beliefs, self-talk, mood, and relationships. Participants interested in participating were informed of their rights and provided online informed consent in accordance with university Institutional Review Board standards. The study received the approval of the University of Valencia ethics committee (H-1488382719361). Inclusion criteria included native Spanish speaking, experience of at least one stable romantic relationship, and possession of a mobile device capable of installing GGRO (available for download via Google Play or the App Store).

Consistent with common practice in OCD-related research, the sample used in the present study comprised nonclinical participants [30]. Like individuals who are clinically diagnosed with OCD, nonclinical participants tend to engage in compulsive behaviors to alleviate distress [31]. Furthermore, taxometric studies of OCD [32] have found that OCD symptoms and obsessive compulsive–related beliefs are best conceptualized as continuous dimensional rather than categorical.

All participants volunteered and were included in a draw for a prize of a dinner for two (valued at 30€). A total of 98 students attended a recruitment seminar wherein they were explained the general procedure of the research. They were then asked to download that app and complete the pretreatment evaluation (Time 1, T1) on Qualtrics [33], which is a secure online survey platform. Emails with the corresponding survey links were sent to participants at Time 2 (T2) and Time 3 (T3). From the 98 students who wanted to take part in the study, one was excluded because he did not have a stable partner in the present or past. The final 97 participants (79 women, 81.4%) were second- and third-year students in the Bachelor of Arts program, with ages ranging from 18 to 65 years (mean 21.56; SD 7.07). The majority (61.9%) reported having a medium socioeconomic status (28.9% below average and 9.3% above average). More than half of the participants (56.3%) were in a romantic relationship at the time of the study (median length, 33 months).

### Study Design

The study was a randomized controlled trial with a crossover design (Figure 1). The intervention was a mobile-delivered cognitive training using GGRO. Participants were randomized to an App first group (iApp, n=51) or a wait list crossover group (dApp, n=46). Participants in the iApp group started using the app immediately (T1) for a period of 15 consecutive days (until T2). They were then requested to stop using the app until the end of the trial. Participants randomized to the dApp group were requested to start using the app at T2 (15 days after the iApp group). They were then requested to use the app (crossover) for the following 15 days. In both groups, participants were instructed to complete 3 levels a day (approximate 3 min a day). The CONSORT-EHEALTH checklist is presented as Multimedia Appendix 1.

### Randomization

Randomization was carried out in a 1:1 ratio and based on a prespecified computer-generated randomization list [34]. Group assignment was performed onsite using the next available number on the randomization list.

### Intervention

**Figure 2:**
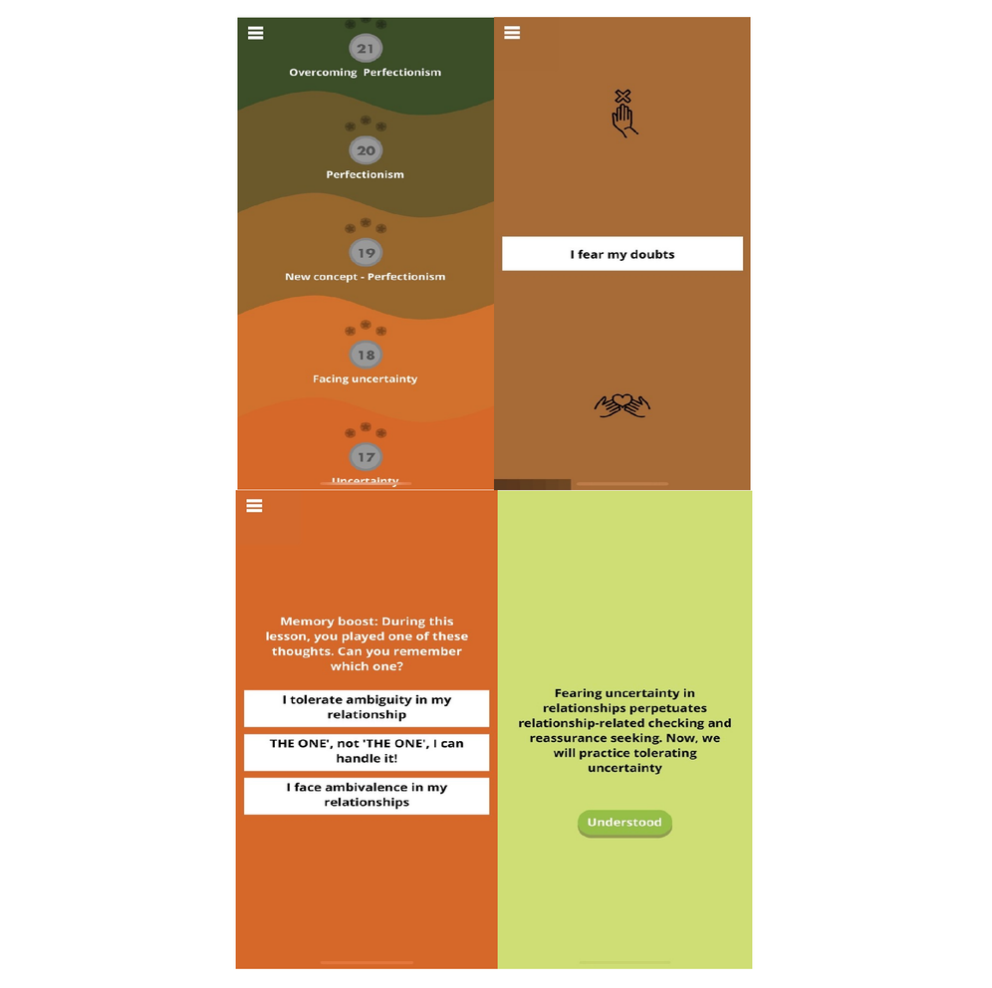
GGRO screenshot.

GGRO was developed by the author GD, an expert in OCD and related disorders, in collaboration with Gur Ilany, a mobile platform developer. This app consists of training exercises intended to help people increase accessibility to functional self-statements that facilitate adaptive interpretations of thoughts, emotions, and events associated with OCD (Figure 2). Users are presented with “blocks” featuring statements such as “I take things as they come” or “Everything can end in a catastrophe.” Users then have to respond to these statements by either embracing them (ie, pulling the “blocks” downwards toward themselves) or rejecting them (ie, throwing the “blocks” upward away from themselves).

Users progressively completed 45 levels dedicated to OCD-related maladaptive beliefs (3 levels per belief) such as dealing with threat, importance of thoughts, and overcoming perfectionism. In this way, the user is exposed to alternative interpretations of the relevant maladaptive belief in each stage, increasing accessibility to functional self-statements that encourage adaptive interpretations for thoughts, emotions, and events (eg, the occurrence of distressing doubts) associated with OCD. For instance, statements challenging perfectionism may include “Mistakes teach me to overcome my fears” and “Imperfect[ion] is human.” Users are also encouraged to adopt approach behavioral strategies (rather than avoidance) including tolerance of negative emotions by responding to statements such as “I can tolerate doubts.”

Following the completion of each level, the user receives feedback, depending on the length of time it took them to complete the level (0 to 3 stars). A short memory-evaluation screen (ie, memory boost) follows this feedback. In this screen, three statements are presented to the user. The user has to recall which of the statements appeared in the level he/she just completed. The correct response results in a “Correct!” message, and an incorrect response is followed by “You’ll get it next time” feedback message. The two types of feedback increase attention to the training and encourage engagement.

The user then progresses to the next level. Three levels address a specific maladaptive belief. Before dealing with a new belief, a screen is presented with the rationale for challenging the specific maladaptive belief. For example, before learning to challenge overestimation of threat, users are presented the statement, “The world can be dangerous, but the tendency to look for danger all the time increases fears and anxieties. Let’s learn to reduce this tendency!” Following completion of six levels pertaining to two beliefs (eg, importance of thoughts and overestimation of threat), users may see an encouraging statement such as “Excellent! Now you’ve learned how to better deal with your thoughts and to better recognize the way you overestimate threat.” Push notifications remind users to use the app each day. Following the completion of 3 levels on a given day, a screen prompting users to stop using the app for that day appears. Users are also advised to train once a day at a preset time rather than in response to distressing thoughts or events. GGRO requires a mobile device with an operating system iOS 7 or above or android 4.2 or above.

### Measures

#### The Obsessive-Compulsive Inventory - Reduced 

The OCI-R [26,27] is a self-report inventory composed of 18 items ranked on a 5-point Likert scale, ranging from 0 (*not at all*) to 4 (*extremely*), which assess OCD symptoms. The OCI-R possesses good internal consistency for the total score (alphas ranging from .81 to .93 across samples) [26]. In our study, the internal consistency for the total scale (Cronbach alpha) was .84 at T1, .83 at T2, and .83 at T3.

#### The Obsessive Beliefs Questionnaire - Short Form

The OBQ-20 [12] is the abbreviated version of the 44-item OBQ - Revised [12]. The OBQ-20 is a self-report questionnaire assessing pan-situational cognitions associated with OCD. It is composed of 20 items ranked on a 7-point scale, ranging from 1 (*disagree very much*) to 7 (*agree very much*). The OBQ-20 has shown satisfactory psychometric properties [35,36]. The internal consistency of the scale as a whole in our sample (Cronbach alpha) was .88 at T1, .93 at T2, and .94 at T3.

#### The Relationship Obsessive-Compulsive Inventory - Short Version

The ROCI - Short version (S) is a shortened version of the ROCI [37], a 12-item measure assessing three dimensions of relationship-centered ROCD symptoms: love for the partner, the “rightness” of the relationship, and the partner’s love for the participant. The ROCI-S consists of 6 items, of which 2 items assess each of the three abovementioned relationship- centered ROCD dimensions (the 2 items showing the highest average loaded on the two original ROCI validation studies; R Moulding, GD, unpublished data, 2019). In an independent sample (n=714; 302 women; mean age 38.73 years; SD 12.65 years), the mean of these six items (ROCI-S total score) showed good reliability (Cronbach alpha=.85) and correlated very highly (r=.97) with the total ROCI total scores. In the current study, the internal consistency (Cronbach alpha) of the mean of all ROCI items was .80 at T1, .83 at T2, and .79 at T3.

#### The Partner-Related Obsessive-Compulsive Symptoms Inventory - 6-Item Version

The PROCSI - 6-item version (Si) (R. Moulding, G.D., unpublished data, 2019) is an abbreviated version of the PROCSI [38], a 24-item measure assessing partner-focused ROCD symptoms. The PROCSI-Si consists of 6 items. The items selected showed the highest correlation of a single item with the relevant subscale in one-half of a randomly split sample (n=356; 151 women; mean age 38.58 years, SD 12.55 years). The mean of these six items (PROCSI-Si total score) showed good reliability (Cronbach alpha=.90) and correlated very highly (r=.98) with the total PROCSI total scores in this sample. The PROCSI-Si total score also showed good reliability scores (Cronbach alpha=.92) and correlated highly (r=.98) with the PROCSI total scores in the independent half of the sample (n=356; 151 females; mean age 38.88 years; SD 12.79 years). The internal consistency of PROCSI-Si (Cronbach alpha) in the current sample was .78 at T1, .83 at T2, and .77 at T3.

#### The Depression, Anxiety, Stress Scale - Short Version

The short version of the Depression, Anxiety, Stress Scale (DASS) [39-42] is a self-report questionnaire that evaluates negative emotional symptoms (depression, anxiety, and stress). The short version consists of 21 items rated on a 4-point scale, ranging from 0 (*did not apply to me at all*) to 3 (*applied to me very much or most of the time*). In this study, only the depression scale (7 items) was used. The DASS scales have been shown to have high internal consistency [42]. The internal consistency of the depression scale (Cronbach alpha) in the current sample was .90 at T1, .90 at T2, and .91 at T3.

#### The Single-Item Self-Esteem Scale

The Single-Item Self-Esteem Scale (SISE) [43] is a self-report measure that determines the extent to which the sentence “I have a high self-esteem” describes participants on a 9-point scale, ranging from 1 (*not very true for me*) to 9 (*very true for me*). The SISE has been found to have high test-retest reliability, criterion validity coefficients above .80 (median=.93 after correcting for unreliability) with the Rosenberg Self-Esteem Scale, and a similar pattern of construct validity coefficients as the Rosenberg Self-Esteem Scale with 35 different constructs [43]. Using longitudinal data, Robins et al [43] estimated the reliability of the SISE to be .75.

### Statistical Analysis

Statistical analyses were performed using Statistical Package for the Social Sciences (SPSS Inc, Chicago, IL). In order to avoid overoptimistic estimates of the efficacy of the training [44], an intention-to-treat analysis using the last-observation-carried-forward method was used [45]. Descriptive statistics were used to report means, SDs, and frequencies. In addition, *t* and *χ*^*2*^ tests were performed to assess differences between groups and in age, relationship duration (in months), sex, socioeconomic level, belief, and symptoms measures (OBQ-20, OCI-R, PROCSI-Si, ROCI, DASS, and SISE). A series of repeated measures analysis of variance with Bonferroni adjustments was performed to evaluate pre-post scores in both study groups. The Effect Size Determination Program [46] was used to calculate Cohen *d* values.

## Results

### Principal Findings

A total of 97 participants met the inclusion criteria and participated in the study. Mean scores for outcome measures and characteristics of the two groups did not differ significantly at baseline (Table 1).

**Table 1:** Descriptive statistics and comparisons between immediate-use App (iApp) group and delayed-use App (dApp) group in sociodemographic variables and outcome measures at baseline.

Characteristics		iApp (n=51)	dApp (n=46)	*t* / *χ^2^*	*df*	*P* value	Cohen *d*
Age (years), mean (SD)		22.88 (9.23)	20.09 (2.73)	1.97	95	.05	0.4
**Gender, %**							
	Men	25.5	10.9	3.42	1	.06	0.37
	Women	74.5	89.1				
**Socioeconomic status, %**							
	Low	3.9	2.2	3.64	3	.30	0.15
	Medium-low	31.4	19.6				
	Medium	52.9	71.7				
	Medium-high	11.8	6.5				
Relationship duration (months), mean (SD)		45.37 (86.96)	19.30 (14.78)	2.00	95	.05	0.4
OCI-R^a^ (score), mean (SD)		1.79 (0.57)	1.78 (0.40)	0.09	95	.92	0.02
OBQ-20^b^ (score), mean (SD)		3.24 (0.96)	3.08 (0.84)	0.84	95	.40	0.17
ROCI-S^c^ (score), mean (SD)		1.76 (0.69)	1.73 (0.61)	0.24	95	.81	0.05
PROCSI-Si^d^ (score), mean (SD)		1.66 (0.57)	1.62 (0.69)	0.29	95	.77	0.06
DASS-D^e^ (score), mean (SD)		1.74 (0.67)	1.53 (0.49)	1.74	95	.08	0.35
SISE^f^ (score), mean (SD)		3.12 (1.09)	3.37 (0.97)	-1.20	95	.23	0.24

^a^OCI-R: Obsessive-Compulsive Inventory - Reduced.

^b^OBQ-20: Obsessive Beliefs Questionnaire-20.

^c^ROCI-S: Relationship Obsessive-Compulsive Inventory - Short version.

^d^PROCSI-Si: Partner-Related Obsessive-Compulsive Symptoms Inventory.

^e^DASS-D: Depression, Anxiety, Stress Scale - Depression.

^f^SISE: Single-Item Self-Esteem Scale.

**Figure 3:**
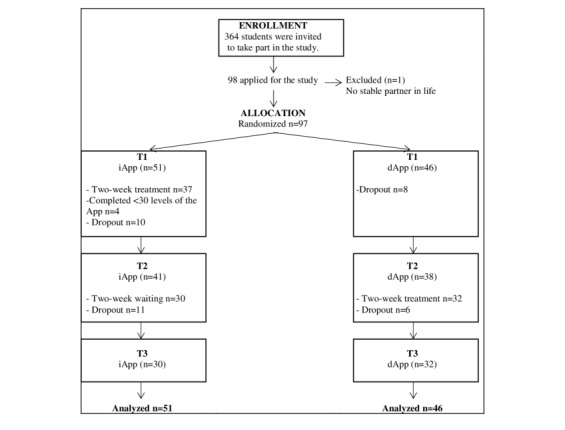
CONSORT flow diagram of participants through the trial.

At 15 days (T2), 79 of 97 participants (81.4%) completed the study and 62 of 97 (65.1%) completed the 15-day follow-up (T3). Participants who dropped out during the study period did not differ in age (*t*_95_*=* 0.58,  *P=*.56), gender (*χ*^*2*^_1_=3.3,  *P=*.79), relationship duration (*t*_95_*=* 0.04,  *P=*.97), or socioeconomic status (*χ*^*2*^_3_=3.3,  *P=*.34) compared to participants who did not drop out (Figure 3).

Tables 2 and 3 present the means and SDs for iApp and dApp participants, respectively, on all measures and testing occasions. All participants used the app for a mean of 14.07 (SD 1.41) days, with a mean of 2.94 (SD 0.37) levels per day. Additionally, the mean of the highest level completed by participants was 40.93 (SD 10.20) levels of the 45 levels. There were no significant differences between the two groups with regard to days used, mean of levels per day, and highest level achieved.

### Between-Group Differences (iApp Group Versus dApp Group)

**Figure 4:**
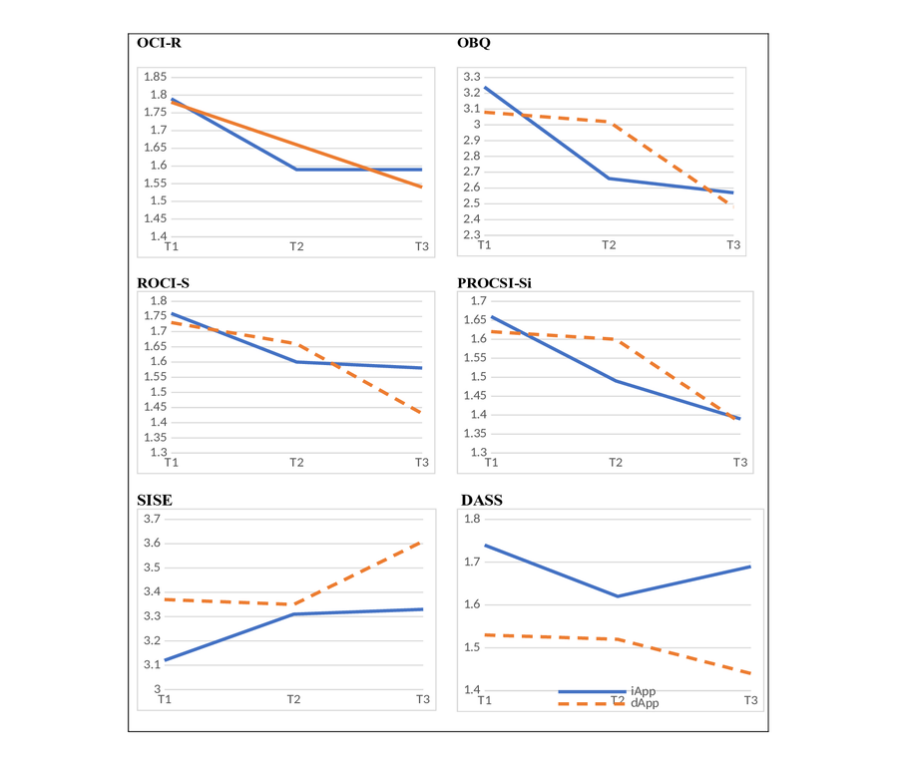
Graphs of the measures across T1, T2, and T3 for iApp and dApp groups. iApp: immediate-use App; dApp: delayed-use App; DASS: Depression, Anxiety, Stress Scale - Short version; OBQ: Obsessive Beliefs Questionnaire; OCI-R: Obsessive-Compulsive Inventory; PROCSI-Si: Partner-Related Obsessive-Compulsive Symptoms Inventory - 6-item version; ROCI-S: The Relationship Obsessive-Compulsive Inventory - Short version; SISE: The Single-Item Self-Esteem Scale.

Analyses of the first two assessment occasions (T1 and T2) revealed significant treatment × repeated measures interactions in OBQ (*F*_1,95_=17.06, *P*<.001, *d*=0.84, PROCSI-Si (*F*_1,95_=4.28, *P*=.04, *d*=0.42), and SISE (*F*_1,95_=4.36, *P*=.04, *d*=0.42). These results indicated that students in the iApp group exhibited fewer OCD-related beliefs, fewer partner-focused ROCD symptoms, and higher self-esteem than their waiting list counterparts on the second assessment occasion (Figure 4).

### iApp Group Within-Group Effects and 15-Day Follow-Up Effects

In the iApp group, we expected pre-post reduction in OCD-related beliefs and symptoms as well as retention of these effects in the follow-up period. Thus, pre-to-final changes were specifically examined via repeated measures analysis of variance between T1 and T3 and between T2 and T3. A significant decline in pre-to-final changes was found in the OBQ, OCI-R, PROCSI-Si, ROCI-S, and SISE scores. Further, the differences found between T1 and T2 were maintained in T3. Moreover, the only statistically significant difference found was in the PROCSI-Si scores that indicated an additional significant improvement between T2 and T3 (Table 2).

**Table 2:** Descriptive statistics and comparisons among periods for the immediate-use App (iApp) group.

Scale	T1 (points), mean (SD)	T2 (points), mean (SD)	T3 (points), mean (SD)	T1 vs T3			T2 vs T3		
				*F*_1,50_	*P* value	*d*	*F*_1,50_	*P* value	*d*
OCI-R^a^	1.79 (0.57)	1.59 (0.50)	1.59 (0.49)	10.87	.002	0.65	0.00	.99	0
OBQ-20^b^	3.24 (0.96)	2.66 (1.10)	2.57 (1.18)	51.39	<.001	1.42	1.20	.28	0.21
ROCI-S^c^	1.76 (0.69)	1.60 (0.62)	1.58 (0.63)	5.65	.02	.47	0.07	.79	0.05
PROCSI-Si^d^	1.66 (0.57)	1.49 (0.55)	1.39 (0.48)	30.00	<.001	1.109	5.98	.02	0.48
DASS-D^e^	1.74 (0.67)	1.62 (0.64)	1.69 (0.69)	0.53	.47	0.14	3.84	.06	0.39
SISE^f^	3.12 (1.09)	3.31 (1.14)	3.33 (1.21)	7.13	.01	.53	0.11	.74	0.06

^a^OCI-R: Obsessive-Compulsive Inventory.

^b^OBQ-20: Obsessive Beliefs Questionnaire - Short form.

^c^ROCI-S: Relationship Obsessive-Compulsive Inventory - Short version.

^d^PROCSI-Si: Partner-Related Obsessive-Compulsive Symptoms Inventory - Six item version.

^e^DASS-D: Depression, Anxiety, Stress Scale-Depression subscale.

^f^SISE: Single-Item Self-Esteem Scale.

**Table 3:** Descriptive statistics and comparisons among periods for the delayed-use App (dApp) group.

Scale	T1 (points), mean (SD)	T2 (points), mean (SD)	T3 (points), mean (SD)	T1 vs T2			T2 vs T3		
				*F*_1,45_	*P*	*d*	*F*_1,45_	*P*	*d*
OCI-R^a^	1.78 (0.40)	1.66 (0.36)	1.54 (0.32)	7.28	.01	0.56	9.09	.004	0.61
OBQ-20^b^	3.08 (0.84)	3.02 (1.03)	2.48 (1.04)	.41	.53	0.13	27.52	.001	1.07
ROCI-S^c^	1.73 (0.61)	1.66 (0.62)	1.43 (0.38)	1.70	.20	0.27	12.52	.001	0.72
PROCSI-Si^d^	1.62 (0.69)	1.60 (0.72)	1.38 (0.50)	.10	.75	0.06	9.41	.004	0.62
DASS-D^e^	1.53 (0.49)	1.52 (0.50)	1.44 (0.37)	.00	.96	0	2.35	.13	0.32
SISE^f^	3.37 (0.97)	3.35 (1.01)	3.61 (0.91)	.07	.78	0.05	6.75	.01	0.53

^a^OCI-R: Obsessive-Compulsive Inventory.

^b^OBQ-20: short form of the Obsessive Beliefs Questionnaire.

^c^ROCI-S: Relationship Obsessive-Compulsive Inventory - short version.

^d^PROCSI-Si: Partner-Related Obsessive-Compulsive Symptoms Inventory - 6-item version.

^e^DASS-D: Depression, Anxiety, Stress Scale-Depression subscale.

^f^SISE: Single-Item Self-Esteem Scale.

### dApp Group Within-Group Effects

In the dApp group, we expected that crossover (ie, use of the app) would be associated with a significant decrease in OCD beliefs and symptom measures. Indeed, within-group differences between T2 and T3 following the crossover indicated significant reductions in the OBQ, PROCSI-Si, ROCI-S, and SISE scores. No differences were found in the DASS scores.

Unexpectedly, participants showed a significant decrease in OCD symptoms (OCI-R) between T1 and T2. Nevertheless, additional significant reduction in OCI-R scores was found between T2 and T3 (Table 3).

## Discussion

Mobile apps based on CBT principles have unique advantages including wide reach, continuous availability, appeal to young people, very low cost, and progress monitoring. Accumulating evidence shows that such apps can lead to significant reductions in psychopathological symptom and maladaptive behaviors [47-49]. The present randomized control study evaluated the efficacy of a mobile app platform named GGRO, which was designed to challenge OCD-related maladaptive beliefs. Consistent with a previous exploratory investigation [24], our results indicated that 15 days of brief daily training using GGRO led to significant reductions in OCD-related beliefs. Moreover, reductions in OCD-related beliefs were maintained for a follow-up period of 2 weeks. These results provide support for the stepped-care approach for OCD [8], suggesting that OCD-related beliefs and symptoms can be reduced using alternative, low-intensity modes of treatment delivery.

Relative to the waitlist control group in our study, individuals using GGRO for 2 weeks showed fewer OCD-related beliefs, fewer ROCD symptoms, and higher self-esteem. Moreover, the change after the training was stably maintained after 15 days (follow-up). Once our waiting list group started using GGRO (after crossover), participants in this group showed a reduction in OCD-related beliefs, OCD, and ROCD symptoms. Interestingly, an exception was partner-focused OC symptoms, which showed further improvements at follow-up, indicating a generalization of results and suggesting that the maladaptive beliefs targeted in GGRO may be particularly relevant to this ROCD presentation. Indeed, GGRO includes levels related to partner-value contingency of self (ie, self-esteem that is overly dependent on the partner’s perceived value [50]), which may be particularly pertinent to partner-focused ROCD symptoms [51]

Unexpectedly, OCD symptoms declined in both the iApp and dApp groups during the initial 2 weeks. Fluctuations in the intensity of student requirements/evaluations may have coincided with the time period of our study and attenuated participants’ OCD symptoms in the waitlist group. Indeed, most of the student data were collected during the mid-semester, a period when students have fewer exams and other evaluations. Importantly, however, the use of GGRO was associated with a further reduction in OCD symptoms following the crossover, thus supporting the efficacy of GGRO in reducing OCD symptoms.

Consistent with a previous study [24], the levels of depression did not show any statistically significant change. Indeed, GGRO was specifically designed to target OCD-related beliefs and symptoms. GGRO was not designed to challenge depression-related maladaptive beliefs per se (eg, hopelessness and helplessness) and therefore does not include content designed to challenge depression-related beliefs. Considering that participants’ depression levels were relatively low, this may explain why the depression symptoms did not show a significant reduction.

Our findings are consistent with those of previous research showing the efficacy of CBT-based apps in cognitive and behavioral change [52,53]. According to these models, commonly occurring intrusive experiences escalate into obsessions due to their catastrophic misinterpretations [12]. Strongly held maladaptive beliefs such as perfectionism, exaggerated importance attributed to the content of thoughts or their occurrence, and low tolerability for uncertainty increase the likelihood of such catastrophic interpretations.

The GGRO app evaluated in this study was designed to challenge maladaptive beliefs associated with OCD. By introducing users to interpretations that are inconsistent with their OCD-related beliefs, adherence to such beliefs was expected to weaken. This, in turn, was expected to reduce catastrophic interpretations of intrusions and decrease OCD symptoms. Indeed, previous findings showed that a change in OCD belief levels among users of GGRO was associated with a reduction in OCD symptoms further from the OCD symptom levels before using GGRO [24]. Consistent with this finding, the results of our study suggest that daily training, which involved active response from users to catastrophic interpretations of intrusions and their alternatives, was shown to lead to a significant reduction in maladaptive beliefs and associated symptoms.

Although the findings of our study are consistent with the expectations, our study has some important limitations. The sample used in our study comprised mainly female students from the general population. Indeed, the prevalence of OCD has been observed to be equal between men and women, or slightly higher in women [3]. Recent reviews support the utility of nonclinical participants in OCD-related research [31]. Moreover, initial evidence suggests that the use of the GG platform with individuals presenting with OCD [54] may reduce OCD-related beliefs and symptoms. Nevertheless, clinical populations may be different from nonclinical participants in symptom-related impairment; the lack of such symptom-related impairment in nonclinical participants may facilitate reduction in OCD-related beliefs and symptoms compared to a clinical population. In addition, the absence of mental disorders was not confirmed by clinical interviews. Future studies may benefit from evaluating the usefulness of GGRO in individuals with OCD.

Previous research using similar methodologies showed dropout rates comparable to ours [55,56]. We also performed intention-to-treat analysis with the last observation carried forward method [45] to prevent overestimation of treatment effects. Nevertheless, care should be taken in the interpretation of our results. Future studies may benefit from the use of additional dropout-reduction strategies (eg, monetary or course credit compensation).

A great majority of mobile apps designed for OCD are oriented toward self-applied therapy and track or guide exposure and response prevention [57,58]. However, their efficacy has not been empirically demonstrated with controlled studies [23,59]. In this regard, this randomized control study furthers our knowledge about the efficacy of alternative CBT-delivery systems for OCD.

GGRO was designed as a brief and easy training platform to challenge maladaptive beliefs and associated interpretations of thoughts and events. As such, this platform could complement traditional CBT interventions as an intersession work or relapse-prevention tool; thus, it is an instrument at the service of the therapist, and not a way to replace CBT. Moreover, this cost-effective and accessible mobile platform could be used in populations at risk of OCD and related disorders to reduce levels of maladaptive beliefs. Future studies should assess the usefulness of similar apps for other symptoms such as body image distress and depression. Indeed, reducing the levels of maladaptive beliefs in at-risk populations using cost-effective, accessible mobile platforms such as the one used in this study may increase resilience to a wide variety of psychological disorders. Furthermore, such a platform may be useful for relapse prevention following treatment.

## Appendix

Multimedia Appendix 1 CONSORT‐EHEALTH checklist (V 1.6.1).

## Abbreviations

CBT: cognitive behavioral therapy
dApp: delayed-use App group
DASS: Depression, Anxiety, Stress Scale - Short version
DASS-D: Depression, Anxiety, Stress Scale - Depression subscale
iApp: immediate-use App group
OBQ-20: Obsessive Beliefs Questionnaire - Short form
OCD: obsessive-compulsive disorder
OCI-R: Obsessive-Compulsive Inventory - Reduced
PROCSI: Partner-Related Obsessive-Compulsive Symptoms Inventory
PROCSI-Si: Partner-Related Obsessive-Compulsive Symptoms Inventory - 6-item version
ROCD: Relationship OCD
ROCI: The Relationship Obsessive-Compulsive Inventory
ROCI-S: The Relationship Obsessive-Compulsive Inventory - Short version
SISE: Single-Item Self-Esteem Scale
